# Supplementary figures and images for: NTDscope: A multi-contrast portable microscope for disease diagnosis
Source: PLOS Glob Public Health. 2026 Feb 12;6(2):e0005937. doi: 10.1371/journal.pgph.0005937 (PMC12900437; doi:10.1371/journal.pgph.0005937)

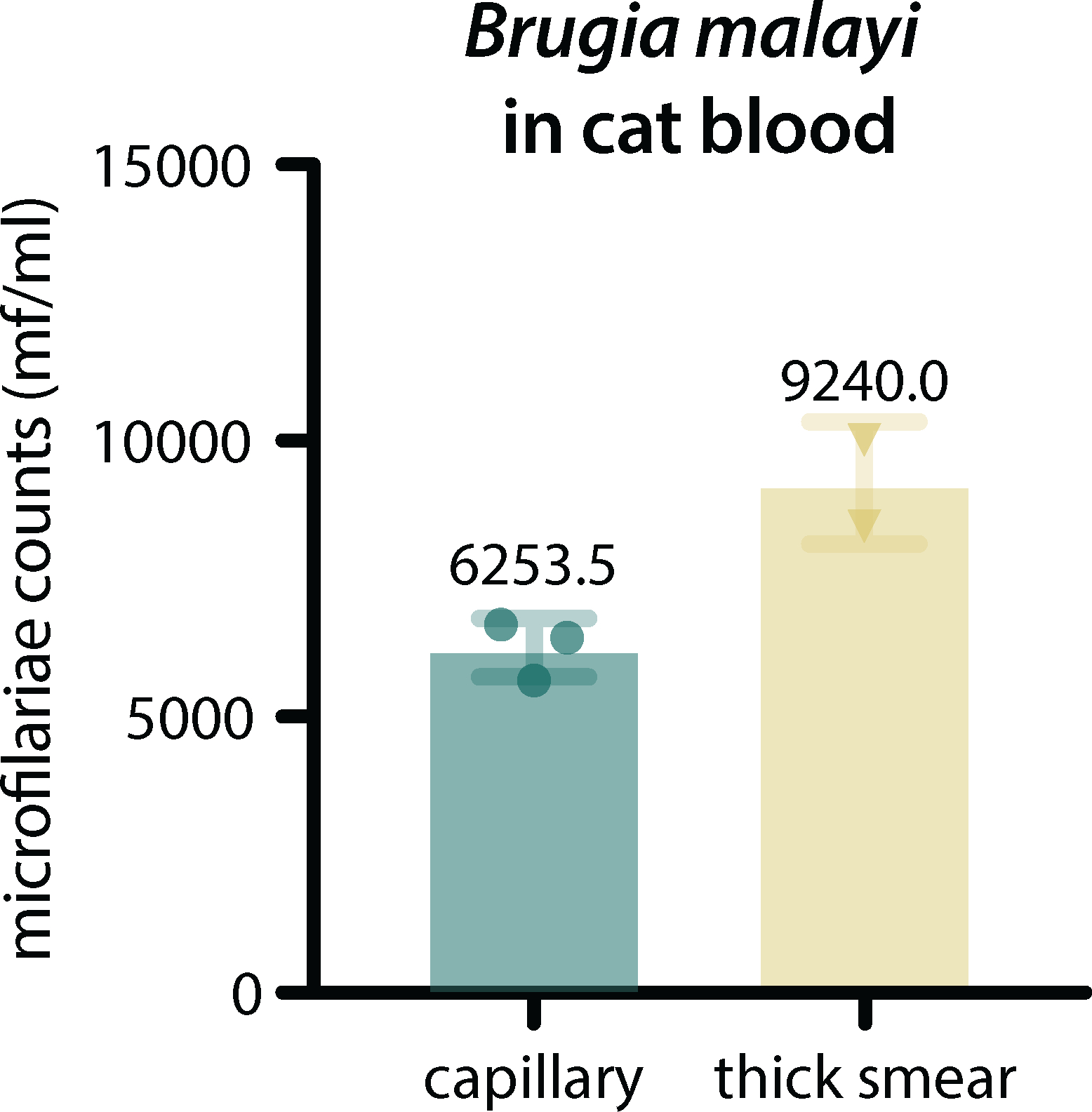

Supplement: S1 Fig — Comparison of B. malayi microfilariae counted manually from videos acquired on the NTDscope and microfilariae counts in calibrated thick smears, from a blood sample of a cat infected with B. malayi. (TIFF) [file pgph.0005937.s001.tif]

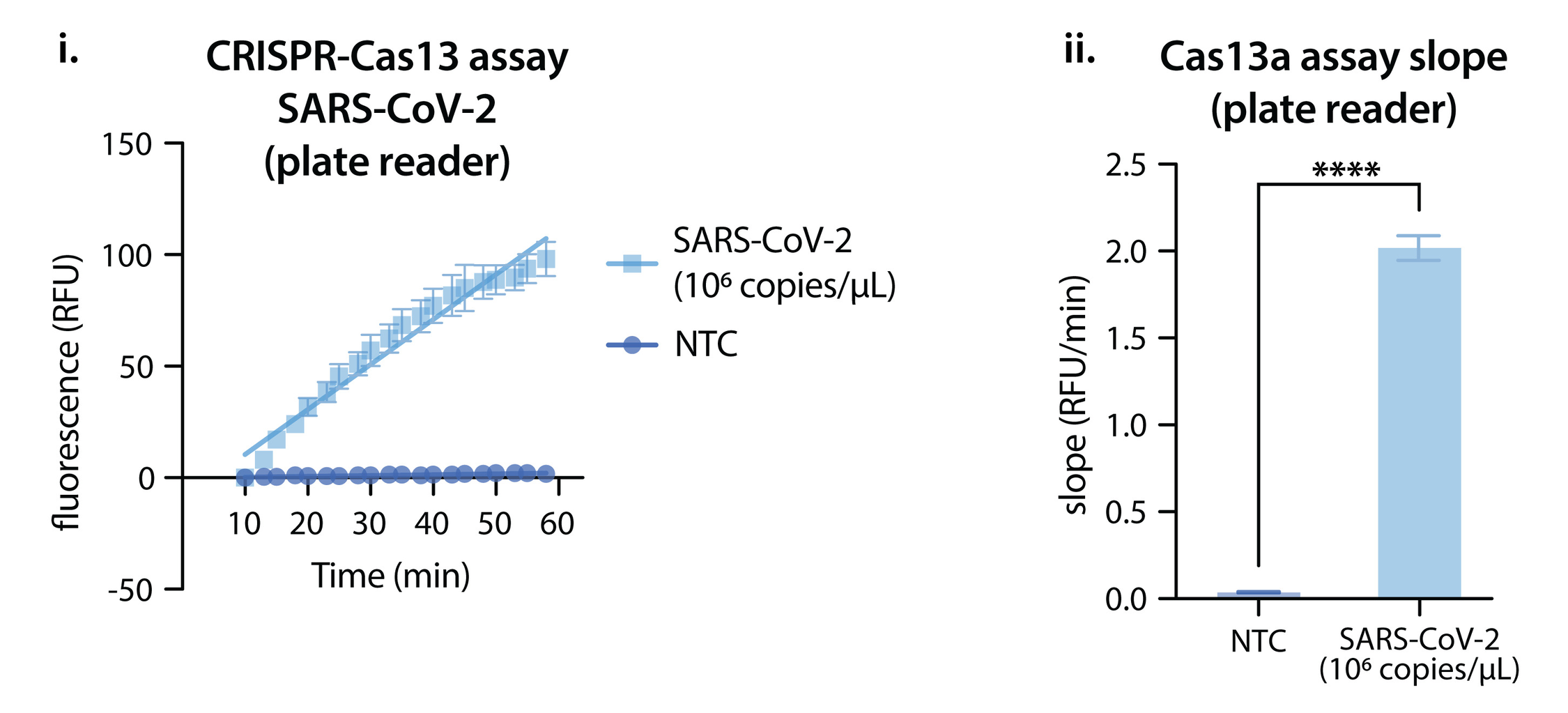

Supplement: S2 Fig — i. Measurement of fluorescence intensity over time for a Cas13 assay with a guide RNA specific to SARS-CoV-2, in the presence (SARS-CoV-2 1e6 copies/μL) and absence (non-target control — NTC) of synthetic SARS-CoV-2 RNA. Fluorescence intensity was measured on a Tecan Spark plate reader during the first 60 minutes of assay time; measurements were acquired every 10 minutes. The data from the first 10 minutes was discarded to allow the assay components to equilibrate to temperature. Experiment performed in triplicate. ii. Slopes of the curves were calculated by performing simple linear regression of the data for each replicate of i. Slopes of the positive samples were compared to the no target RNA controls (NTC) using an unpaired t-test. Data are expressed as mean ± standard error of the mean (SE). is p < 0.05; ** is p < 0.01; *** is p < 0.001; **** is p < 0.0001. Data was processed and visualized using GraphPad Prism 10. (TIFF) [file pgph.0005937.s002.tif]
