# Supplementary material for: NTDscope: A multi-contrast portable microscope for disease diagnosis
Source: PLOS Glob Public Health. 2026 Feb 12;6(2):e0005937. doi: 10.1371/journal.pgph.0005937 (PMC12900437; doi:10.1371/journal.pgph.0005937)
Supplement: S1 Checklist — (PDF) [file pgph.0005937.s006.pdf]

Inclusivity in global research

Ethical considerations, permits and authorship

This section is applicable to all research types.

Provide details as to who granted permissions and/or consent for the study to take place in the Methods section of your manuscript. This should include the names of **all** ethics boards, governmental organizations, community leaders or other bodies that provided approval for the study. If individuals provided approval refer to these people by their role or title but do not list their name(s).

The details on who granted permissions and consent for each study are listed in the Materials and Methods section of our manuscript (page number 6).

If there were any deviations from the study protocol after approval was obtained please provide details of these changes in the Methods section of your manuscript.

There were no deviations from the study protocol after approval was obtained.

Did this study involve local collaborators that are residents of the country where the research was conducted or members of the community studied? If you do not have any authors from said communities, please provide an explanation for this below.

Yes, they are included as authors.

Everyone listed as an author should meet PLOS' criteria for authorship and all individuals who meet these criteria should be included in the author byline, rather than the acknowledgements. For further information please see the journal's Authorship Policy.

Everyone listed as an author meets PLOS' criteria for authorship.

Human subjects research (e.g. health research, medical research, cross-cultural psychology)

Did you obtain written informed consent from a representative of the local community or region before the research took place? How did you establish who speaks for the community? Details of written informed consent obtained from study participants should be reported separately in the Methods section of your manuscript.

Local collaborators (listed as authors in the manuscript) first communicated with local health systems (health districts, community health personnel, and community leaders) to inform them of the proposed research. Community representatives then communicate with their community members before conducting any study activities. This ensures transparent information and inclusion of communities in all research activities. Details of the informed consent obtained from study participants are reported in the Methods section of our manuscript.

How did members of the local community provide input on the aims of the research investigation, its methodology, and its anticipated outcome(s)?

In each community, all eligible individuals were invited to a central point (health facility or chieftaincy). An introductory speech was given by a team member and translated into the local language by community health personnel. All potential participants were given the opportunity to ask questions before making the decision to participate or not.

When engaging with the local community, how did you ensure that the informed consent documents and other materials could be understood by local stakeholders?

Information about the study and consent forms were translated into the local language by community health personnel, who were available to answer questions from participants before they signed a consent form.

Will the findings of the research be made available in an understandable format to stakeholders in the community where the study was conducted (e.g. via a presentation, summary report, copies of publications, etc.)? Please provide details of how this will be achieved.

The NTDscope is a diagnostic tool intended to accelerate the work of researchers in the countries where the study was carried out, and we will be providing copies of publications to our local partners and other researchers who are interested in the technology. That is one of our motivations for publishing this work in an open access journal. Our local co-authors will use the results of this paper to explain the technology to communities that may benefit from it, and will use the device capabilities to advance the research. Where required, the key findings of the current paper have already been translated into the local official language in a report format and submitted to the ethical committee and health authorities of the health districts where the data have been collected.

Non-human subjects research using specimens/ animals collected as part of the study, or those housed in archival collections. Examples include archaeology, paleontology, botany and zoology.

Some samples were obtained from animals specifically raised for research purposes. *S. mansoni* schistosoma eggs were extracted from the livers of infected hamsters provided by the Schistosomiasis Resource Center of the Biomedical Research Institute. *Brugia malayi* in cat blood was provided by the NIH/NIAID Filariasis Research Reagent Resource Center for distribution through BEI Resources.

Did the permission you obtained from a local authority to perform the study include an agreement on access to outputs and benefit sharing? This may include procedures to enable fair distribution of the benefits and resources arising from the research performed. Please include any details of Prior Informed Consent and Benefit Sharing Agreements obtained. These may be required by field-specific regulations, for example the Convention on Biological Diversity (CBD) and the associated Nagoya Protocol.

If the material used in your study was imported, please A) provide the year it was imported and B) indicate whether permits were obtained to import/export the materials used, C) provide details of any permits obtained. If this information is not available, please indicate this.

If you used archival specimens, please state how the material used in your study was acquired by the institute it is held in and provide details of any permits obtained for the original excavations/ sample collection. If this information is not available, please indicate this.

How was the potential cultural significance of the materials collected in your study to local communities considered in your research design? Were Indigenous peoples and/or local researchers and institutions involved with archaeological excavations / collection of specimens? If so, please provide a description of their involvement.

If your manuscript includes photographs of human remains please indicate whether authors obtained permission from descendants or affiliated cultural communities to do so.
